# Improved Resistance Switching Stability in Fe-Doped ZnO Thin Films Through Pulsed Magnetic Field Annealing

**DOI:** 10.1186/s11671-017-1949-4

**Published:** 2017-03-09

**Authors:** Hongtao Xu, Changjin Wu, Zhao Xiahou, Ranju Jung, Ying Li, Chunli Liu

**Affiliations:** 1 Department of Physics and Oxide Research Center, Hankuk University of Foreign Studies, Yongin, 449-471 Korea; 2 Laboratory for Microstructures/School of Materials Science and Engineering, Shanghai University, 149 Yanchang Road, Shanghai, 200072 People’s Republic of China; 3 Department of Electrophysics, Kwangwoon University, Seoul, 139-701 Korea

**Keywords:** RRAM, ZnO, Fe doping, Magnetic annealing

## Abstract

Five percent of Fe-doped ZnO (ZnO:Fe) thin films were deposited on Pt/TiO_2_/SiO_2_/Si substrates by a spin-coating method. The films were annealed without (ZnO:Fe-0T) and with a pulsed magnetic field of 4 T (ZnO:Fe-4TP) to investigate the magnetic annealing effect on the resistance switching (RS) behavior of the Pt/ZnO:Fe/Pt structures. Compared with the ZnO:Fe-0T film, the ZnO:Fe-4TP film showed improved RS performance regarding the stability of the set voltage and the resistance of the high resistance state. Transmission electron microscopy and X-ray photoelectron spectroscopy analyses revealed that the ZnO:Fe-4TP film contains more uniform grains and a higher density of oxygen vacancies, which promote the easier formation of conducting filaments along similar paths and the stability of switching parameters. These results suggest that external magnetic fields can be used to prepare magnetic oxide thin films with improved resistance switching performance for memory device applications.

## Background

As a potential next-generation nonvolatile memory, transition metal oxide (TMO)-based resistance random access memory (RRAM) has been studied intensively during the last decade and has attracted increasing interest because of its low power consumption, high operation speed, high endurance, and simple structure [1–3]. Zinc oxide (ZnO), which is a well-known oxide semiconductor, has also been widely studied because of its resistance switching (RS) behaviors [4–8]. ZnO-based RRAM devices have been reported to show an ultrafast programming speed of 5 ns, an ultrahigh ON/OFF ratio of 10 [7], a long retention time of more than 10^7^ s, and high reliability at elevated temperatures [2, 5]. However, several problems need to be elucidated before achieving practical device applications. One of the issues is minimizing the dispersion of memory switching parameters, such as the resistance values of the low- and high-resistance states (LRS and HRS, or ON and OFF) and the switching voltages from the HRS to LRS (set voltages, V_set_) and vice versa (reset voltages, V_reset_) [6, 9]. The dominant cause of the oscillation of the switching parameters is the intrinsic random nature of the formation of defect-dominated conducting filaments (CFs) during the switching process [10]. Many attempts, such as doping impurity elements [11–13] and interfacial engineering [14, 15], have been reported to be effective for controlling the location of the CFs and therefore stabilizing the switching parameters.

In this work, we report the effect of annealing in a magnetic field on the RS properties of Fe-doped ZnO thin films. A magnetic field can be used not only to study the physical properties but also to synthesize magnetic materials or modify their properties [16]. The application of an external magnetic field during material synthesis can affect the structural and magnetic properties of the prepared materials [17–22]. Annealing transition metal (TM)-doped ZnO nanoparticles with a high-pulsed magnetic field has been reported to improve the magnetic properties and increase oxygen defects [18, 19, 23, 24], which motivated us to study the magnetic annealing effect on the RS behaviors. In this work, we annealed Fe-doped ZnO (ZnO:Fe) thin films under a pulsed magnetic field of 4 T and determined that the magnetic field annealing process has a dramatic stabilizing effect on the switching parameters of Pt/ZnO:Fe/Pt structures.

## Methods

Five percent of Fe-doped ZnO thin films were prepared on Pt(111)/TiO_2_/SiO_2_/Si substrates using a spin-coating method. Zinc acetate [Zn(CH_3_COO)_2_ · 2H_2_O] and iron nitrate [Fe(NO_3_)_3_ · 9H_2_O] were used as the precursors, and 2-methoxyethanol (HOCH_2_CH_2_OCH_3_) and monoethanolamine (H_2_NC_2_H_4_OH, MEA) were used as the solvent and stabilizer, respectively. The precursor chemicals were first dissolved completely in 2-methoxyethanol, then mixed together with the addition of MEA. The obtained mixture solution with a total metal ion concentration of 0.5 M was stirred at 60 °C for 0.5 h, then aged for 24 h before deposition on the substrate. The spin-coating process was performed at 3000 rpm for 30 s, followed by heating at 100 °C for 10 min to evaporate the solvent and pre-annealing at 400 °C for 10 min to exclude organic residuals. The spin-coating process was repeated several times to obtain a thickness of ~100 nm. The deposited films were annealed at 650 °C for 1 h in air, either with or without a 4-T pulsed magnetic field, labeled as ZnO:Fe-0T and ZnO:Fe-4TP, respectively.

The crystalline orientation and microstructure of the thin films were characterized by X-ray diffraction (XRD) with Cu Kα radiation and transmission electron microscopy (TEM). The chemical states were characterized by X-ray photoelectron spectroscopy (XPS), performed with a monochromatic Al Kα X-ray source (hv = 1486.6 eV) at an energy of 15 kV/150 W. The spot size was 400 μm (Theta Probe AR-XPS System, Thermo Fisher Scientific). Top Pt electrodes with dimensions of 90 × 90 μm [2] were deposited using e-beam evaporation to fabricate the RRAM devices, and the current-voltage (I-V) characteristics of the RRAM devices were measured using a semiconductor device parameter analyzer (Agilent B1500A).

## Results and Discussion

**Fig. 1:**
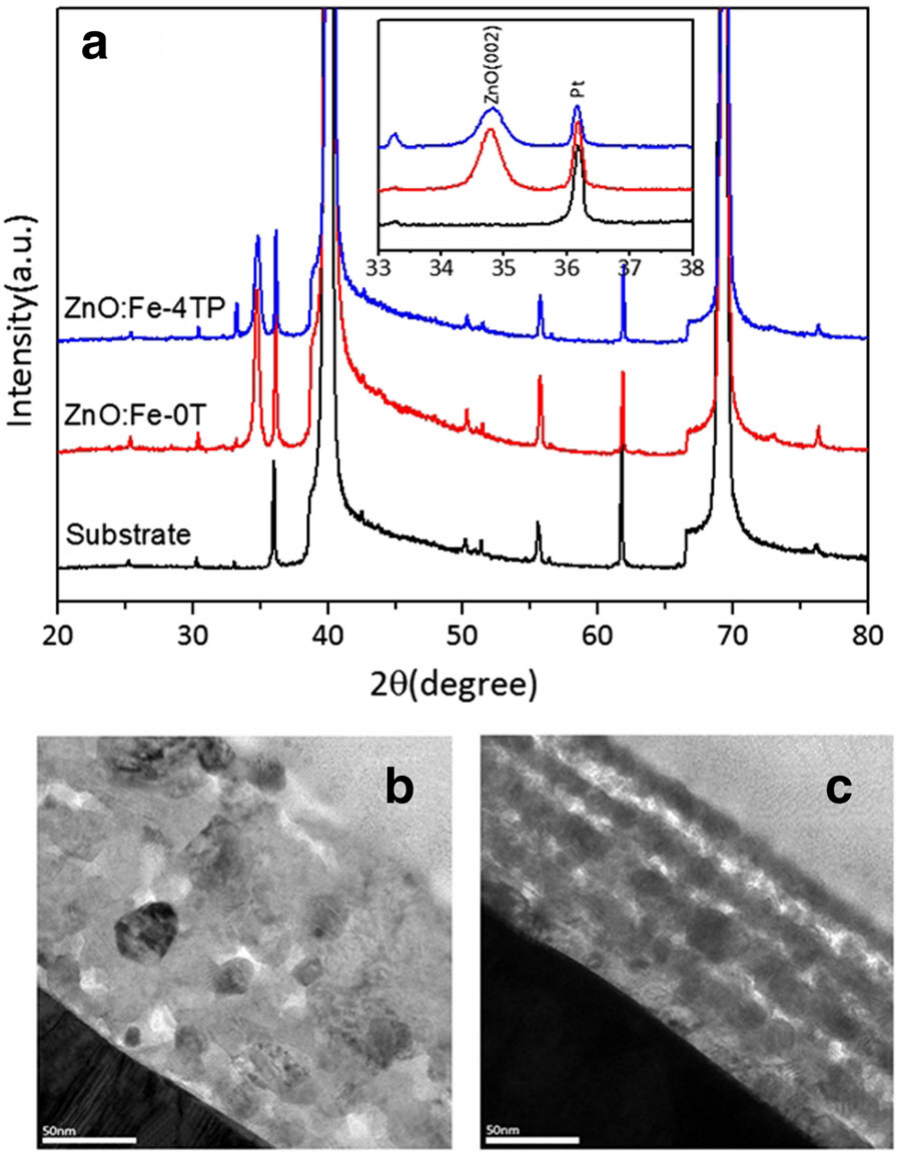
**a** XRD patterns of ZnO:Fe-0T and ZnO:Fe-4TP films. **b, c** The cross-section TEM images of ZnO:Fe-0T and ZnO:Fe-4TP films, respectively

Zn_0.95_Fe_0.05_O films annealed with and without a magnetic field were revealed to have a hexagonal wurtzite structure preferentially oriented in the [002] direction, as shown in Fig. 1a. Despite the similarity in the XRD spectra, the TEM images of the ZnO:Fe-0T and ZnO:Fe-4TP showed quite different characteristics. In the cross-section images of the ZnO:Fe-0T thin film (Fig. 1b), non-uniform grains with different sizes and irregular positions were observed, which is quite different from the uniformly distributed grains in the ZnO:Fe-4TP thin film, shown in Fig. 1c. The crystallinity of the ZnO:Fe-4TP film seems to be improved by the magnetic field annealing process, which is similar to the results reported for hydrothermally prepared TM-doped ZnO nanoparticles [25–27]. The better crystallinity observed after magnetic annealing can be attributed to the reduced temperature gradient and more homogeneous nucleation rate induced by the magnetic field [25].

**Fig. 2:**
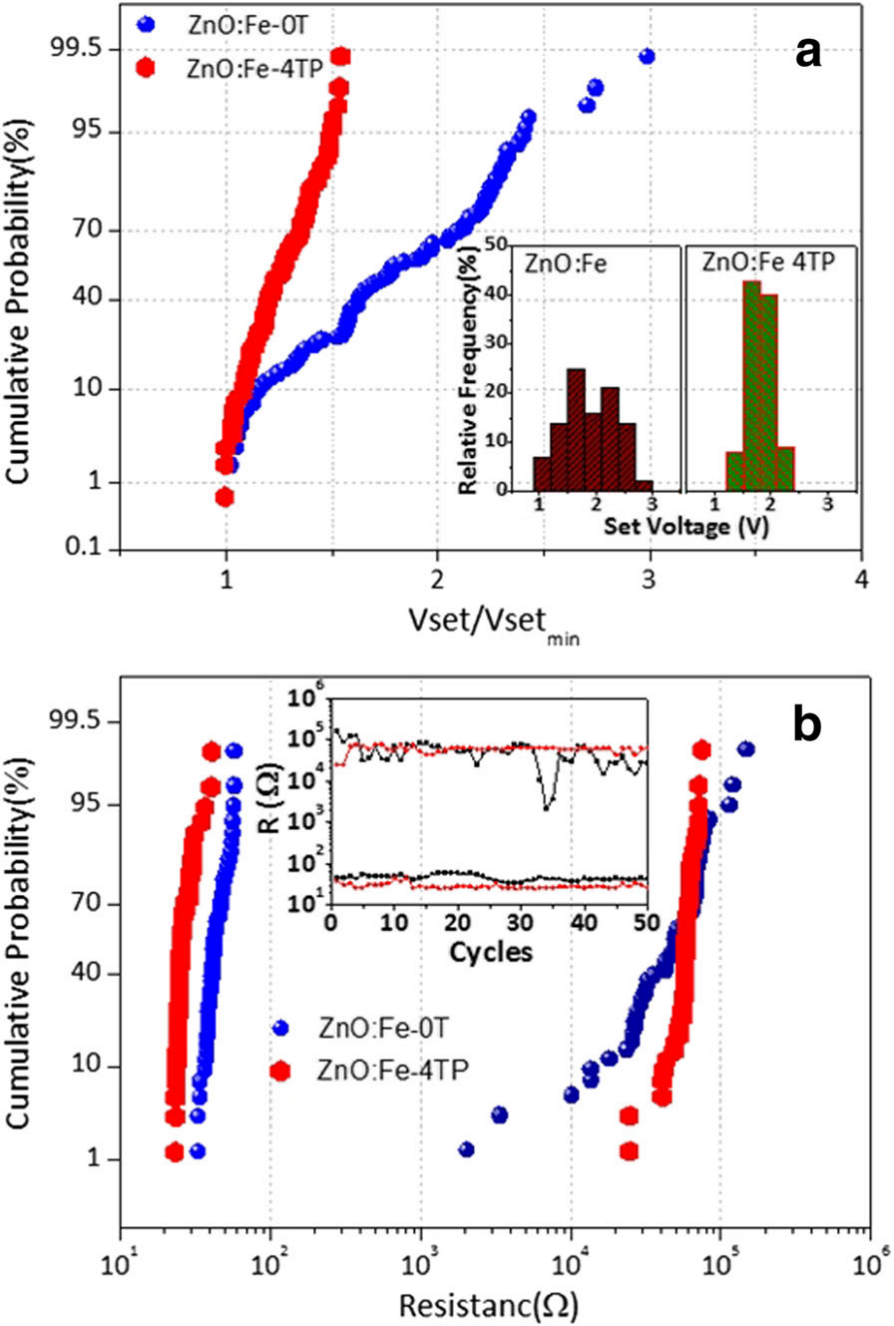
**a** The cumulative probability of the set voltages of ZnO:Fe-0T and ZnO:Fe-4TP films. The *insets* show the relative frequency of the set voltage for both devices. **b** The cumulative probability of resistance in the HRS and LRS for ZnO:Fe-0T and ZnO:Fe-4TP films. The *inset* shows the endurance properties of both films for 50 cycles

Both RRAM devices fabricated with the ZnO:Fe-0T film and the ZnO:Fe-4TP film showed repeated unipolar resistance switching behaviors. Because of the high initial resistance (>10^8^ Ω), a forming process was necessary to induce the switching process by generating the CFs in the oxide layer. The distribution of switching parameters, however, showed quite obvious differences between the two types of devices. In Fig. 2a, the cumulative distributions of V_set_ in the two types of devices are compared. To ensure statistical correctness, a total of 100 data points measured from 5 devices with 20 switching cycles each are plotted for each type of device. To clearly compare the data, V_set_ was first normalized with respect to the minimum V_set_ among the same type of device then plotted on the X axis. The relative frequency probabilities of V_set_ of both films are shown in the inset figures of Fig. 2a. Obviously, V_set_ of Pt/ZnO:Fe-4TP/Pt varied within a much narrower range (1.42 ~ 2.18 V) compared with that of Pt/ZnO:Fe-0T/Pt (1.06 ~ 3.18 V), indicating that the stability of V_set_ was improved significantly by applying the magnetic field during annealing of the ZnO:Fe thin film.

The cumulative distributions of the HRS and LRS of the Pt/ZnO:Fe/Pt devices read at 0.1 V are shown in Fig. 2b. A significant enhancement of stability for the HRS was observed in the ZnO:Fe-4TP film. For the ZnO:Fe-0T film, the resistance varied from 2 to 200 kΩ for the HRS, and the LRS was approximately 40 Ω. In contrast, for the ZnO:Fe-4TP film, the resistance of the HRS remained near 50 kΩ, and the LRS was approximately 25 Ω. The inset shows the endurance properties of both films from 50 switching cycles; it is clear that the resistance of the HRS in the ZnO:Fe-4TP film is more stable than that of the ZnO:Fe-0T film. Because the HRS resistance directly affects the value of V_set_ during the following set process, the stabilized HRS resistance values were consistent with the stabilization in V_set_ shown in Fig. 2a [28]. Additionally, the lower LRS resistance in Pt/ZnO:Fe-4TP/Pt implied that stronger CFs existed in this device.

**Fig. 3:**
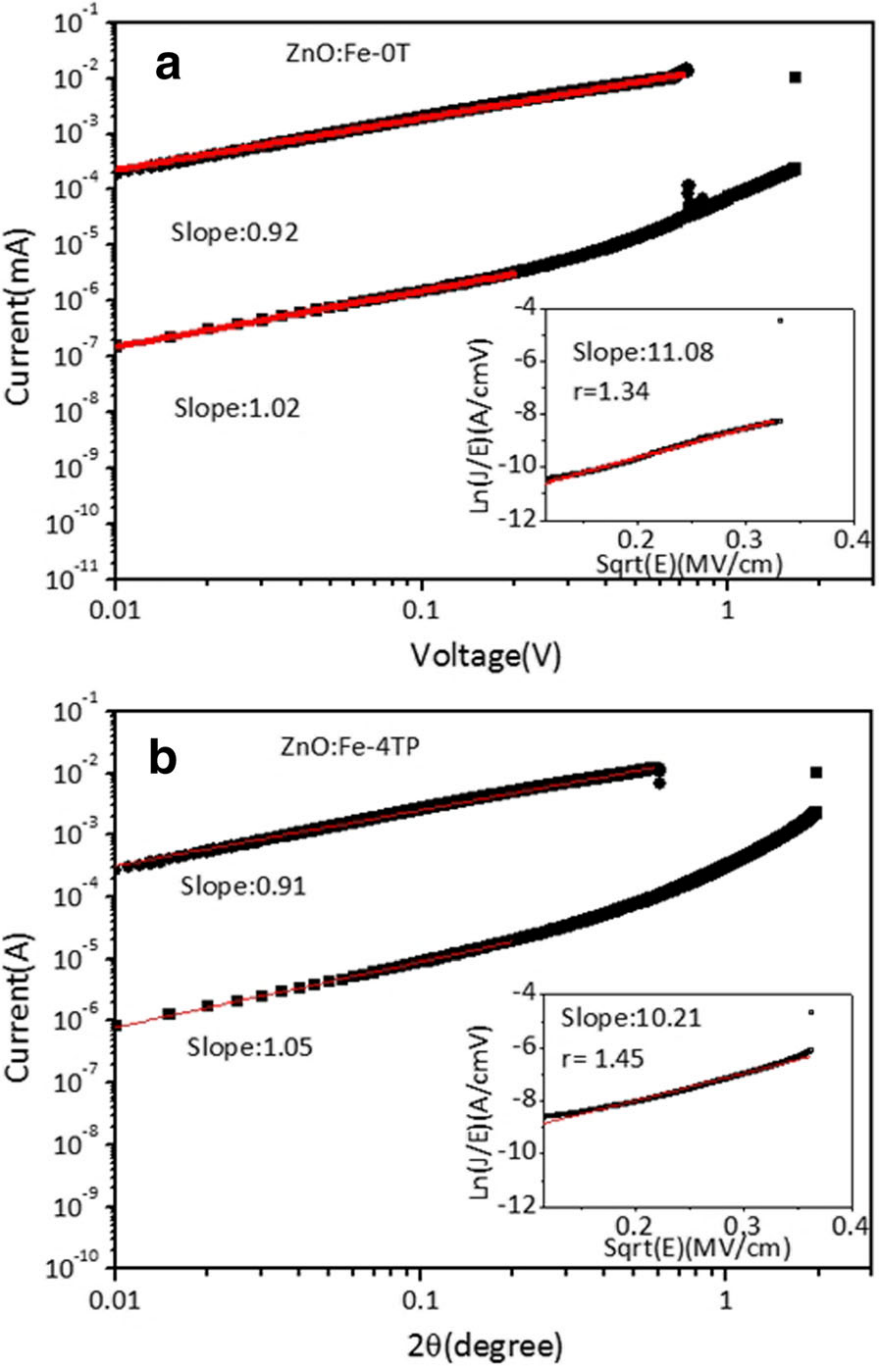
The logarithmic plots of the I-V curves of the HRS and LRS for **a** ZnO:Fe-0T and **b** ZnO:Fe-4T. The *inset panels* show the fitting of the I-V curves at high electric fields using the PF emission mechnism

The stability of switching parameters was first analyzed through the conductive mechanism by fitting the typical I-V curve of Pt/ZnO:Fe/Pt devices, as shown in Fig. 3. The double-log scale I-V curves from Pt/ZnO:Fe-0T/Pt (Fig. 3a) and Pt/ZnO:Fe-4TP/Pt (Fig. 3b) indicate obvious Ohmic characteristics for the LRS and HRS in the low-electric field region for both devices. The high-electric field regions of the HRS, on the other hand, can be fitted with Poole-Frenkel (PF) emission using the relationship $\ln \left( J/ E\right)\propto \left(\sqrt{q^3/\pi {\varepsilon}_0{\varepsilon}_r}/ rkT\right)\bullet \sqrt{E}$ [27], where *q* is the electric charge, *ε*_*r*_ is the dynamic dielectric constant, *ε*_*0*_ is the permittivity of free space, *k* is Boltzmann’s constant, *T* is the temperature, and *r* is a constant with a value between 1 and 2. The insets of Fig. 3a, b show that with the refractive index *n* = (*ε*_*r*_^1/2^) for pure ZnO (~2.00) and the slope of the ln(*J/E*) vs. *E* ^1/2^ curve, the estimated values of *r* are approximately 1.34 and 1.45 for Pt/ZnO:Fe-0T/Pt and Pt/ZnO:Fe-4TP/Pt, respectively. Because the PF emission describes the hopping of carriers via trapped states excited by an electric field, a value of *r* larger than 1 implies the existence of traps in the thin film [29–31]. Accordingly, the higher *r* value in Pt/ZnO:Fe-4T/Pt indicates that the total number of traps increased in the ZnO:Fe-4TP film as compared with the ZnO:Fe-0T film [32].

**Fig. 4:**
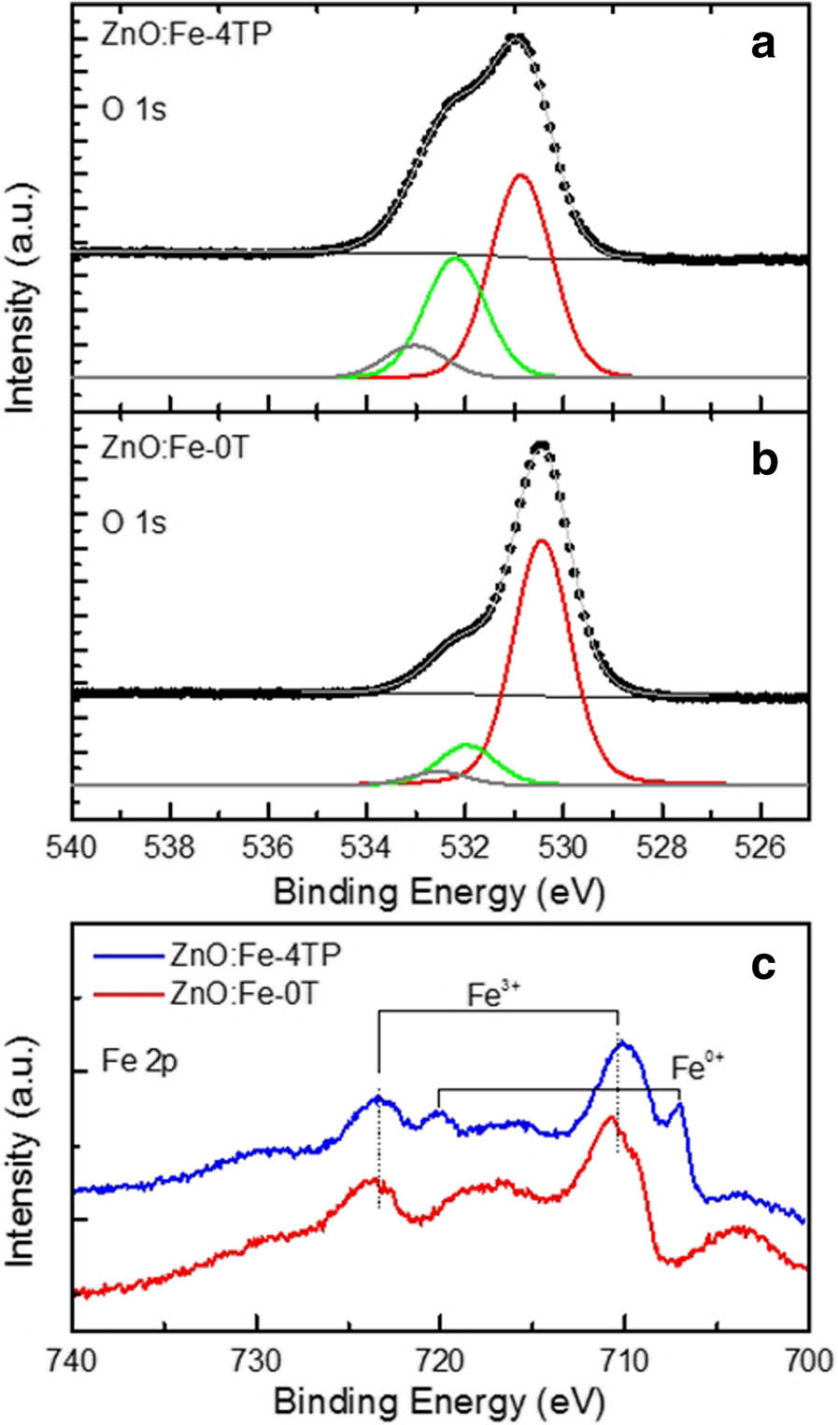
**a** and **b** The XPS spectra of O 1s in ZnO:Fe-0T and ZnO:Fe-4TP films shown together with the deconvolution results. **c** The XPS spectra of Fe 2p in ZnO:Fe-0 T and ZnO:Fe-4TP films

Because various kinds of defects, including oxygen vacancies, can all act as trapping centers in oxide thin films, analysis of the composition and valence states of elements in ZnO:Fe thin films could help to understand the improved RS properties caused by magnetic annealing. In this vein, XPS characterization of ZnO:Fe-0T and ZnO:Fe-4TP thin films was carried out, and the spectra of Fe and O ions are shown in Fig. 4.

The O 1s spectra from the two films showed quite different profiles, as shown in Fig. 4. The deconvolution results contain three peaks located around 533, 532, and 530 eV, which can be attributed to surface adsorbed O, oxygen deficiency, and lattice oxygen [33], respectively. Obviously, there is more oxygen deficiency in ZnO:Fe-4TP films. More interestingly, the Fe 2p spectra revealed that Fe^3+^ ions are dominant in both films (peak located at 711 eV). Additionally, metallic Fe (peak located at 707 eV) is also observed in the ZnO:Fe-4TP film. Although the valence state of Fe should be divalent Fe^2+^ if it is substituted into a defect-free ZnO crystal lattice, the appearance of Fe^3+^ implies the existence of Zn vacancies in our spin-coated ZnO thin films. It has been reported that in Fe-doped ZnO nanocrystals, Fe^3+^ appeared when Zn vacancies were present near the substitutional sites to neutralize the charge imbalance [34]. A similar phenomenon has also been reported for the observation of Cr^3+^ ions in Cr-Mn-doped ZnO under magnetic annealing [35].

**Fig. 5:**
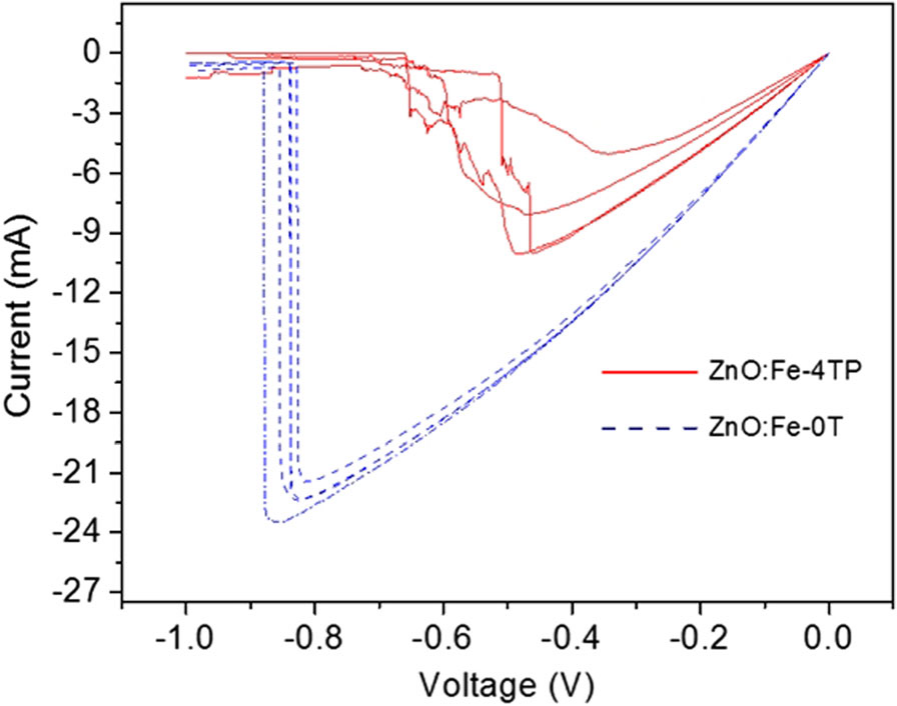
Reset switching behaviors for Pt/ZnO:Fe-0T/Pt and Pt/ZnO:Fe-4T/Pt devices under negative bias voltage. Both devices were set with a positive bias

The existence of metallic Fe and its effect on the switching properties were further revealed from Fig. 5. When Pt/ZnO:Fe-0T/Pt and Pt/ZnO:Fe-4T/Pt devices were set and reset both under a positive bias, i.e., positive set and positive reset, the reset voltage values and the reset currents are similar (data not shown). However, when a negative bias was applied for the reset process, i.e., positive set and negative reset, the reset voltage and reset current are much smaller for Pt/ZnO:Fe-4T/Pt than for Pt/ZnO:Fe-0T/Pt (Fig. 5). This observation can be understood considering the existing of metallic Fe, which could be converted to Fe ions under an external bias and participate in the formation of conducting filaments. When opposite bias voltages were used for the set and reset process, the Fe ions in the conducting filaments may have been pushed back to their original location. This can assist in the dissolution of the conducting filaments, resulting in lower reset parameters and gradually changed resistance [36].

**Fig. 6:**
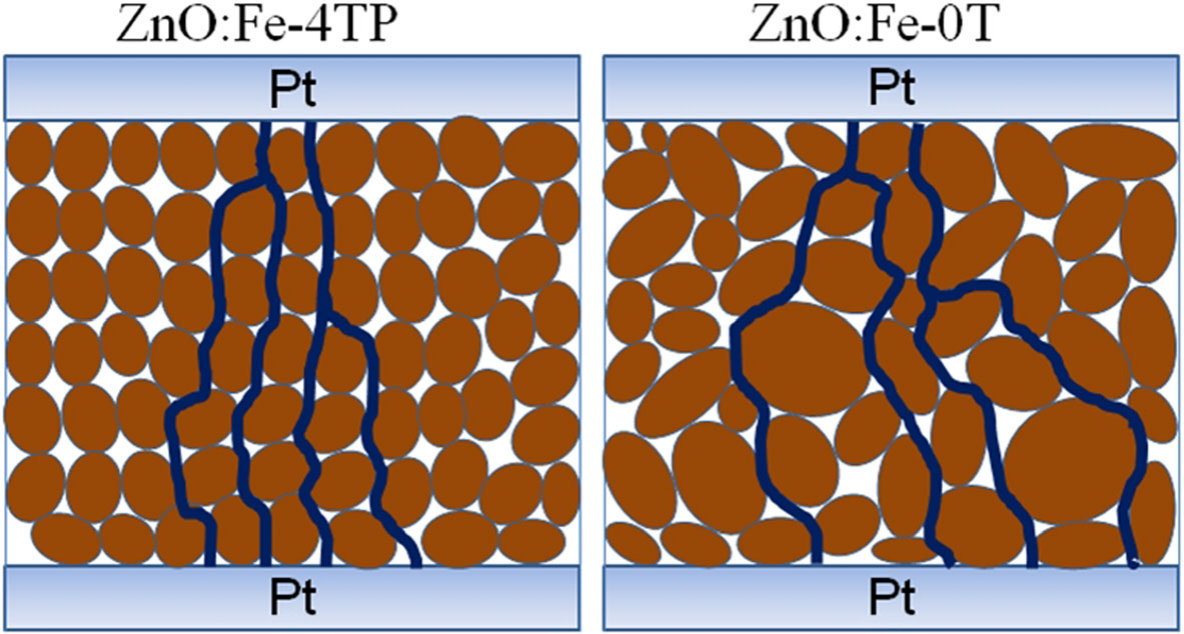
A schematic showing the crystalline structure and conducting filaments in ZnO:Fe-0T and ZnO:Fe-4TP films

The above electrical and physical property characterizations indicate that magnetic annealing affects not only the crystalline structure but also the defects contained in the oxide thin film. Although the reason for the formation of metallic Fe and more oxygen deficiencies in magnetic annealed ZnO:Fe thin films needs further investigation, it is quite clear from our results that more defects, including zinc vacancies, oxygen vacancies, and metallic Fe, are available in ZnO:Fe-4TP thin films as trapping centers. Because the filaments are composed of defects such as oxygen vacancies or metal ions, an increased amount of these defects in ZnO:Fe-4TP makes the formation of conducting filaments more likely. The increase in oxygen vacancies agrees well with the larger value of *r* in Fig. 3. Furthermore, the TEM image revealed that the grain boundaries in the ZnO:Fe-4TP thin film are more regular. Because it has been generally considered that extended defects such as grain boundaries provide diffusion paths for defects in oxide thin films to migrate and connect together to form conducting filaments, the location and shape of the conducting filaments in ZnO:Fe-4TP should be more regular and uniform in each switching circle, compared with the irregular and branch-shaped grain boundaries in ZnO:Fe-0T films (Fig. 6), which is consistent with the lower LRS resistance observed in Fig. 2. The magnetic annealing process enhanced both factors in conducting filament formation, i.e., the fast diffusion path and amount of defects; therefore, better switching stability can be achieved.

## Conclusions

In summary, Fe-doped ZnO thin films were synthesized by the spin-coating method, and the films were annealed with and without a 4-T pulsed magnetic field. The Pt/ZnO:Fe/Pt structures were prepared to investigate the effect of magnetic annealing on the RS behaviors of ZnO:Fe thin films. Unipolar resistance switching was observed in all samples. Detailed analysis of the switching behaviors revealed that the ZnO:Fe-4TP thin film showed better performance regarding a quite stable set voltage and resistance in the HRS. SEM and TEM indicated the grain size became smaller and more uniform in theZnO:Fe-4TP film and the grain boundary is more clear and regulated. Based on the XPS characterization, the improved switching characteristics of the ZnO:Fe-4TP film were attributed to the increased amount of oxygen vacancies, which provided easier and more stable formation of conducting filaments. Our results suggest that by applying a 4-T pulsed magnetic field during the preparation of Fe-doped ZnO films, the resistance switching performance of the set voltage can be improved greatly.

## Abbreviations

CF: Conducting filament
HRS: High-resistance state
I-V: Current-voltage
LRS: Low-resistance state
PF: Poole-Frenkel
RRAM: Resistance random access memories
RS: Resistance switching
TEM: Transmission electron microscopy
TM: Transition metal
V_reset_: Reset voltage
V_set_: Set voltage
XPS: X-ray photoelectron spectroscopy
XRD: X-ray diffraction
